# Quantitative evaluation of interstitial pneumonia using 3D-curved high-resolution CT imaging parallel to the chest wall: A pilot study

**DOI:** 10.1371/journal.pone.0185532

**Published:** 2017-09-28

**Authors:** Hiroyasu Umakoshi, Shingo Iwano, Tsutomu Inoue, Yuanzhong Li, Shinji Naganawa

**Affiliations:** 1 Department of Radiology, Nagoya University Graduate School of Medicine, Nagoya, Aichi, Japan; 2 Imaging Technology Center, Fujifilm Corporation, Tokyo, Japan; Washington State University, UNITED STATES

## Abstract

**Objectives:**

To quantify the imaging findings of patients with interstitial pneumonia (IP) and emphysema using three-dimensional curved high-resolution computed tomography (3D-cHRCT) at a constant depth from the chest wall, and compare the results to visual assessment of IP and each patient’s diffusing capacity of the lungs for carbon monoxide (DLco).

**Methods:**

We retrospectively reviewed the axial CT findings and pulmonary function test results of 95 patients with lung cancer (72 men and 23 women, aged 45–84 years) with or without IP, as follows: non-IP (n = 47), mild IP (n = 31), and moderate IP (n = 17). The 3D-cHRCT images of the lung at a 1-cm depth from the chest wall were reconstructed automatically using original software; total area (TA), high-attenuation area (HAA) >-500 HU, and low-attenuation area (LAA) <-950 HU were calculated on a workstation. The %HAA and %LAA were calculated as follows: $\%HAA=\frac{HAA}{TA}\times 100$, and $\%LAA=\frac{LAA}{TA}\times 100$.

**Results:**

The %HAA and %LAA respective values were 3.2±0.9 and 27.7±8.2, 3.9±1.2 and 27.6±5.9, and 6.9±2.2 and 25.4±8.7 in non-IP, mild IP, and moderate IP patients, respectively. There were significant differences in %HAA between the 3 groups of patients (*P*<0.001), but no differences in %LAA (*P* = 0.558). Multiple linear regression analysis revealed that %HAA and %LAA were negatively correlated with predicted DLco (standard partial regression coefficient [b*] = -0.453, *P*<0.001; b* = -0.447, *P*<0.001, respectively).

**Conclusions:**

The %HAA and %LAA values computed using 3D-cHRCT were significantly correlated with DLco and may be important quantitative parameters for both IP and emphysema.

## Introduction

Interstitial pneumonia (IP) is a condition in which inflammation and fibrosis diffusely affect the pulmonary interstitium and parenchyma [1]. IP and emphysema often occur in combination with primary lung cancer, because these diseases are strongly associated with smoking [2]. Patients with combined pulmonary fibrosis and emphysema (CPFE), which is a unique disorder of the lungs that comprises upper lung emphysema and lower lung fibrosis, are especially at high risk for lung cancer and have a poor prognosis [3]. Therefore, the preoperative assessment of patients with lung cancer for these coexisting diseases is important. Pulmonary function testing (PFT) is usually performed for evaluation of preoperative respiratory function. However, the percent vital capacity (%VC) and ratio of forced expiratory volume in 1 second to forced vital capacity (FEV_1_/FVC) are often normal in patients with CPFE, whereas diffusion capacity of the lungs for carbon monoxide (DLco) is low [3].

High-resolution computed tomography (HRCT) of the chest using a slice thickness ≤1 mm is an excellent method for revealing emphysema and IP, and is essential for their visual assessment [4]. High-speed multi-detector row CT can scan the whole lung with thin slices and provide high resolution images of the whole lung. It enables more precise evaluation of both emphysema and IP on three-dimensional CT (3D-CT). In addition, computer-aided diagnosis (CAD) applied to 3D-CT scans enables quantitative analysis of emphysema [5, 6]. Studies have shown that a percent low attenuation area (%LAA) of less than -950 Hounsfield units (HU) on 3D-CT was significantly correlated with results of PFT, especially with (FEV_1_/FVC) [7, 8].

On the other hand, the development of quantitative CAD for IP has been more challenging [9]. One reason for the difficulty is that CAD cannot automatically differentiate a diffuse pulmonary lesion from large hilar vessels or bronchi. To develop CAD for IP, we focused our attention on the fact that most of idiopathic and smoking-related IP lesions characteristically spread into the peripheral lung located immediately below the chest wall, and we created a CAD software program that uses serial HRCT images to automatically produce a three-dimensional, curved multi-planar reconstruction of the lung at a constant depth from the chest wall. We named it 3D curved HRCT (3D-cHRCT), tentatively. Since this novel CAD only evaluates an image depicting the zone of peripheral lung where there are no pulmonary vessels or bronchi, quantification of IP is feasible.

The purpose of this pilot study was to attempt to quantify the extent of IP and emphysema in patients with lung cancer using 3D-cHRCT at a constant depth from the chest wall, and compare the results to visual assessment of IP and the results of PFT, especially pulmonary diffusion capacity.

## Materials and methods

This retrospective study was approved by our institutional review board at Nagoya university graduate school of medicine, and informed consent was waived (approved No. 636–3).

### Patient selection

We reviewed the CT images and reports stored in a picture archiving and communication system (PACS) of patients with lung cancer identified from a database, who underwent surgical resection from April 2006 to December 2011. At least 2 radiologists had evaluated the preoperative CT images and provided formal reports on the findings of lung cancer and coexisting diseases such as emphysema and interstitial pneumonia. For this study, all CT reports of the identified patients were investigated, and cases with reports of findings suspicious for emphysema or IP were extracted from the PACS. We selected 51 cases with primary lung cancer and IP, and collected their medical history including smoking history, past history of connective tissue disease, and the results of preoperative pulmonary PFT which included DLco, from the clinical records. The time between each patient’s CT examination and PFT was less than 2 months. Three patients with synchronous bilateral lung cancer were excluded. Finally, a total of 48 patients were included in the study. A total of 47 control patients, who had unilateral lung cancer and no IP findings on CT were also included in the study.

### Pulmonary function testing

Preoperative PFT was performed using a flow-sensing spirometer (FUDAC-77; Fukuda Denshi Co. Ltd., Tokyo, Japan) within 2 months after CT image acquisition. The results of PFT included percent predicted (%)forced vital capacity (FVC), %VC, FEV_1_/ FVC, percent predicted (%)DLco, and DLco divided by alveolar volume (%DLco/V_A_).

### HRCT image acquisition and visual assessment

All preoperative CT scans were performed using a 64-multidetector row CT scanner (Aquilion; Toshiba Medical, Tokyo, Japan) in the craniocaudal direction during inspiratory apnea without contrast enhancement using the following scan parameters: x-ray tube voltage, 120 kVp; automatic tube-current; gantry rotation speed, 0.5 sec; and beam collimation, 64 × 0.5mm. Axial thin-section CT images of whole lung were reconstructed with a slice thickness of 0.5-mm at the same increment using a high-spatial frequency algorithm. Iterative reconstruction technique was not used. All reconstructed CT images were transferred to the picture archiving and communication system (PACS) at our hospital.

The axial HRCT images of extracted cases were reviewed independently by two chest radiologists with 3 and 20 years of experience reading thoracic CT scans. Both radiologists thereafter solved all disagreements by consensus reading of images, and the presence of emphysema and IP was finally diagnosed. Emphysema was defined as well-demarcated areas of decreased attenuation as compared with contiguous normal lung, and that were marginated by a very thin (<1 mm) wall or no wall, and/or that showed multiple bullae (>1 cm), with upper zone predominance. The severity of emphysema was evaluated visually using the Goddard classification. A score of 1 represents destruction of 1%–25% of the lung by emphysema; a score of 2, destruction of 26%–50% of the lung; a score of 3, destruction of 51%–75% of the lung; and a score of 4, destruction of 76%–100% of the lung[10]. Fibrosis was also identified on CT images as reticular opacities with peripheral and basal predominance, honeycombing, architectural distortion, and/or traction bronchiectasis or bronchiolectasis; focal ground-glass opacities and/or areas of alveolar condensation could be associated but should not be prominent. The same chest radiologists visually evaluated the severity of fibrosis on axial HRCT images. The overall extent of these abnormalities was determined for each entire lung using a four-point scale (0 = no involvement; 1 = 1%–25% involvement; 2 = 26%–50%; 3 = 51%–75%; and 4 = 76%–100%) [11]. The patients in this study were all pending surgical procedure, and very few patients scored 3 or 4. Therefore, we classified all cases into 3 categories as follows; score 0, non-IP; score 1, mild IP; and score2-4, moderate IP. And each radiological diagnosis of definite Usual Interstitial Pneumonia (UIP) pattern, possible UIP pattern, and inconsistent with the UIP pattern was made according to the 2011 American Thoracic Society (ATS)/European Respiratory Society (ERS)/Japanese Respiratory Society (JRS)/Latin American Thoracic Association (ALAT) diagnostic criteria for UIP/IPF [12]. We also evaluated the existence of pleural effusion or extensive atelectasis.

### Quantification of emphysema and IP by CAD

**Fig 1:**
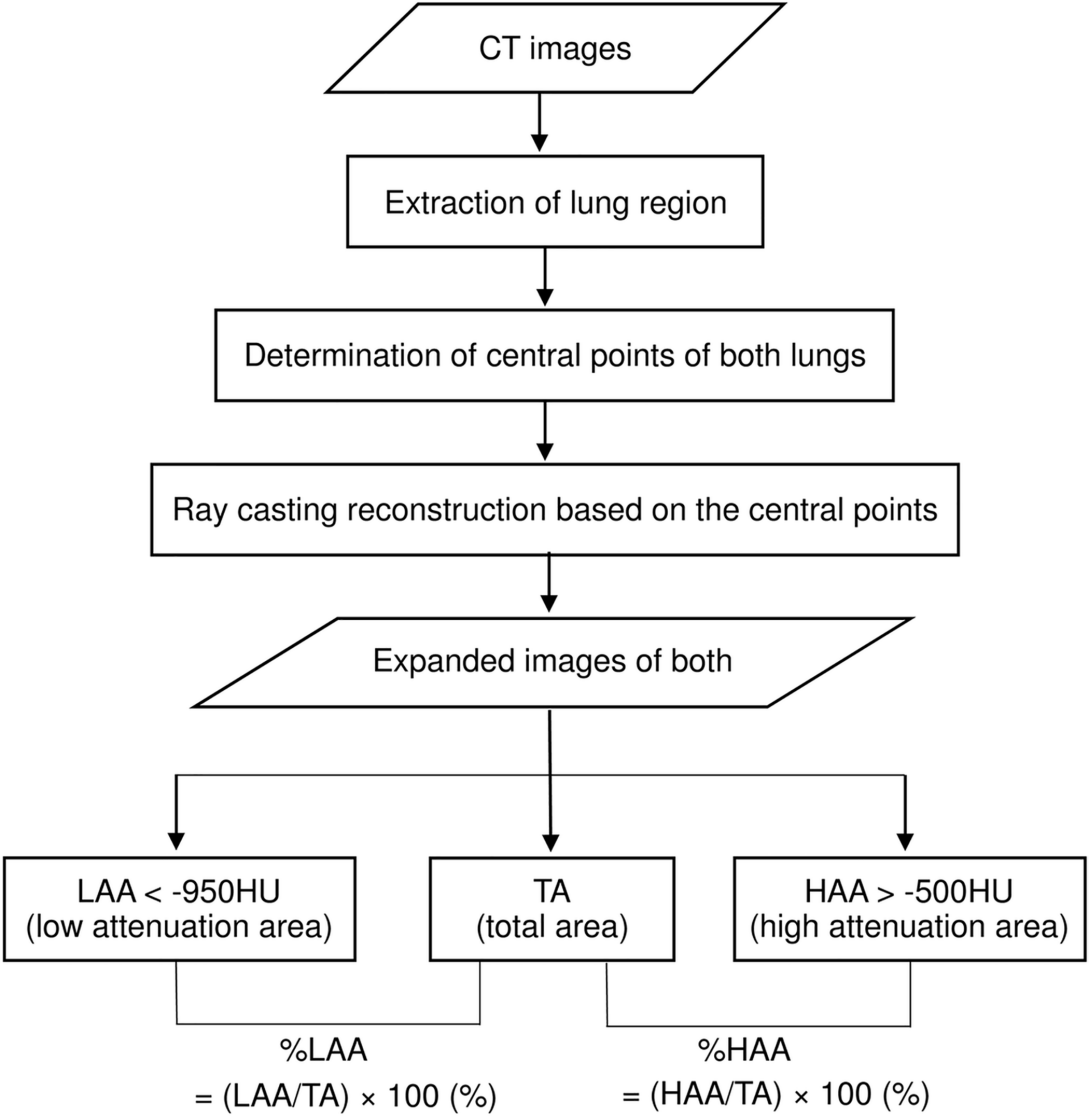
Schematic diagram of 3D-cMPR imaging at a constant depth from the chest wall and assessment. Overall schematic diagram of the three-dimensional, curved high-resolution CT (3D-cHRCT) image at a constant depth from the chest wall and how it is used for assessment. Total area (TA), low attenuation area (LAA [< -950 HU]), and high attenuation area (%HAA) [> -500 HU]), which were obtained from 3D-cHRCT images, were computed on a workstation using a novel CAD program.

**Fig 2:**
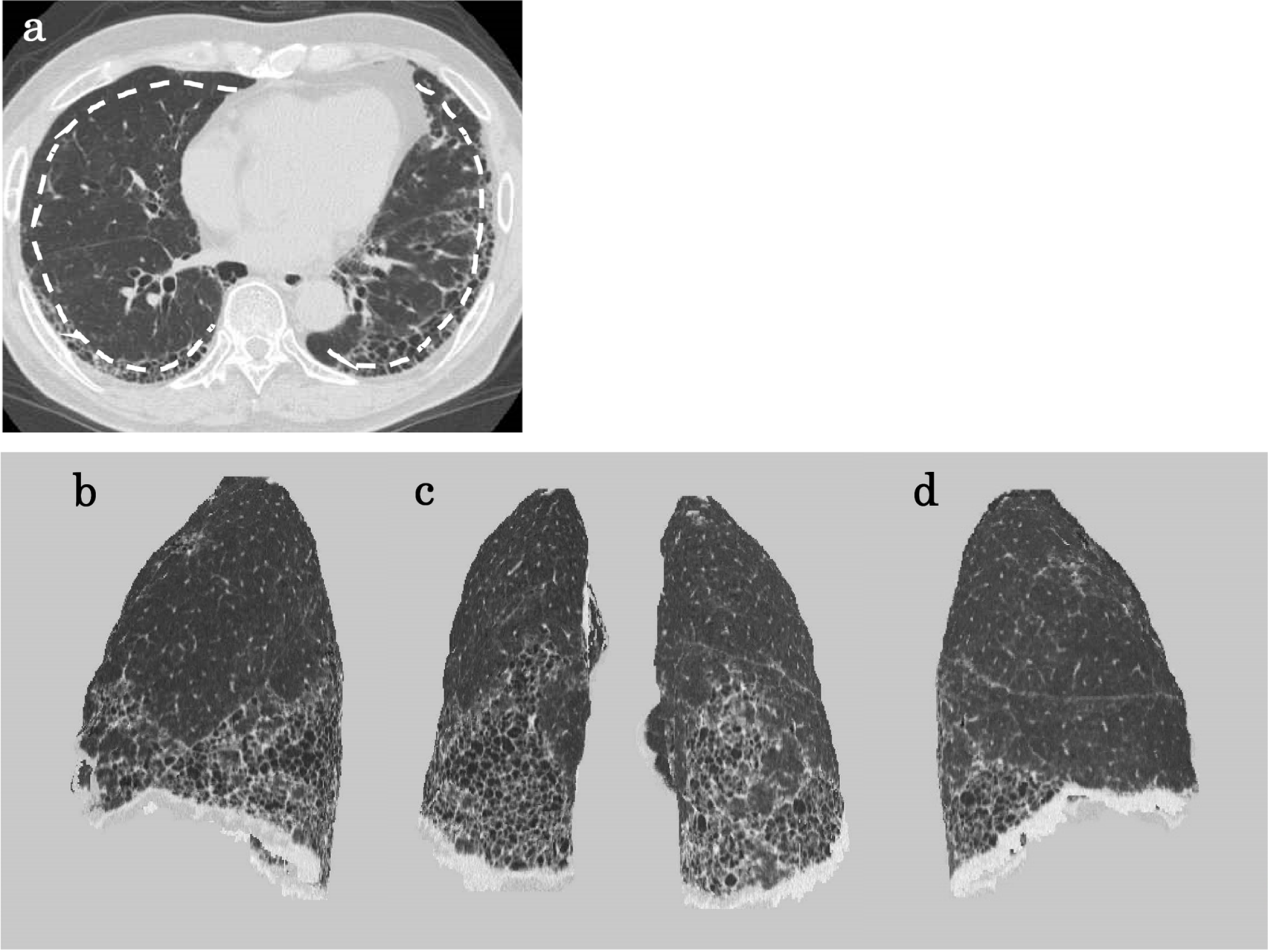
A patient with clinical and radiological typical usual interstitial pneumonia. A patient with clinical and radiological typical usual interstitial pneumonia. (a) Axial HRCT image and dashed line indicating 1-cm depth from the chest wall; multiple-view 3D-cHRCT images showing the 3D distribution of IP infiltrates; (b) left lateral view, (c) posterior view, (d) right lateral view.

The axial HRCT imaging data of the study patients were transferred to the 3D-workstation that automatically used our original software to reconstruct each 3D-cHRCT image of the lung at a constant 1-cm depth from the chest wall. The 1-cm depth from the chest wall was selected from the results of a preliminary experiment (S1 Table). Figs 1 and 2 show the overall scheme of the 3D-cHRCT reconstruction procedure and examples of 3D-cHRCT images, respectively. To eliminate the effects the lung cancer lesion or areas of peripheral inflammation, we analyzed the HRCT data from the contralateral tumor-free lung.

The total area (TA) of the lung and low-attenuation area (LAA) (<−950 HU), which denotes emphysema, on the 3D-cHRCT images were calculated by the CAD program on the workstation. The percentage of low-attenuation area (%LAA) was defined as follows:

$$\%LAA=\frac{LAA}{TA}\times 100\;(\%)$$

In a similar fashion, the percentage of high-attenuation area (%HAA) (higher than the threshold value [>-500 HU]), which denotes IP, was defined as follows:

$$\%HAA=\frac{HAA}{TA}\times 100\;(\%)$$

We used -500 HU as the threshold value based on our preliminary experiment (S1 Table).

**Fig 3:**
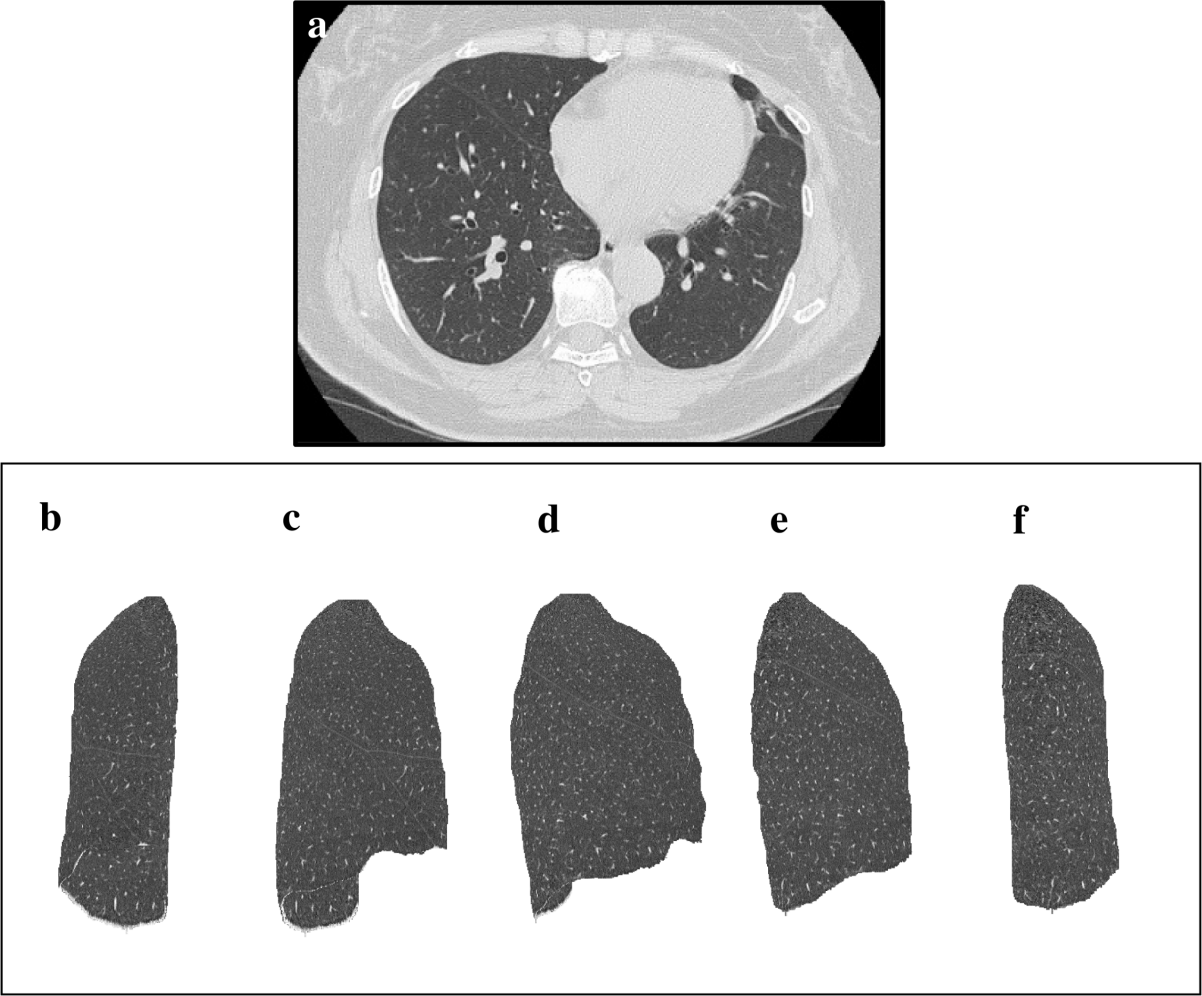
An example of HRCT axial view and 3D-cHRCT images of lung without IP. An example of HRCT axial view and 3D-cHRCT images of lung without IP. (a) HRCT axial view and 3D-HRCT image of the left lung; (b) anterior view, (c) anterolateral view, (d) right lateral view, (e) posterolateral view, (f) posterior view. %HAA values of this case was 2.53%.

**Fig 4:**
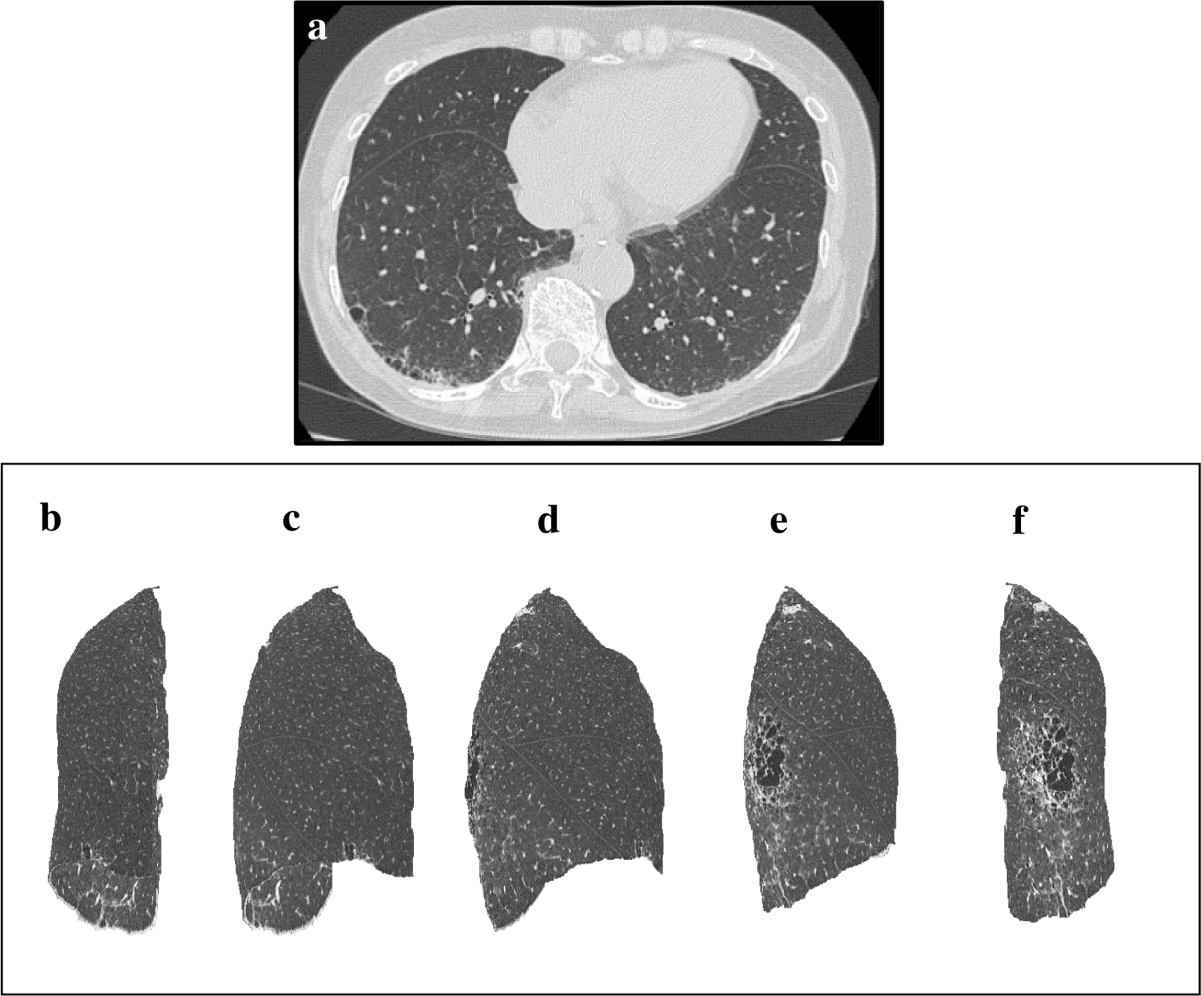
An example of HRCT axial view and 3D-cHRCT images of lung with mild-IP. An example of HRCT axial view and 3D-cHRCT images of lung with mild-IP. (a) HRCT axial view and 3D-HRCT image of the left lung; (b) anterior view, (c) anterolateral view, (d) right lateral view, (e) posterolateral view, (f) posterior view. %HAA values of this case was 6.74%.

**Fig 5:**
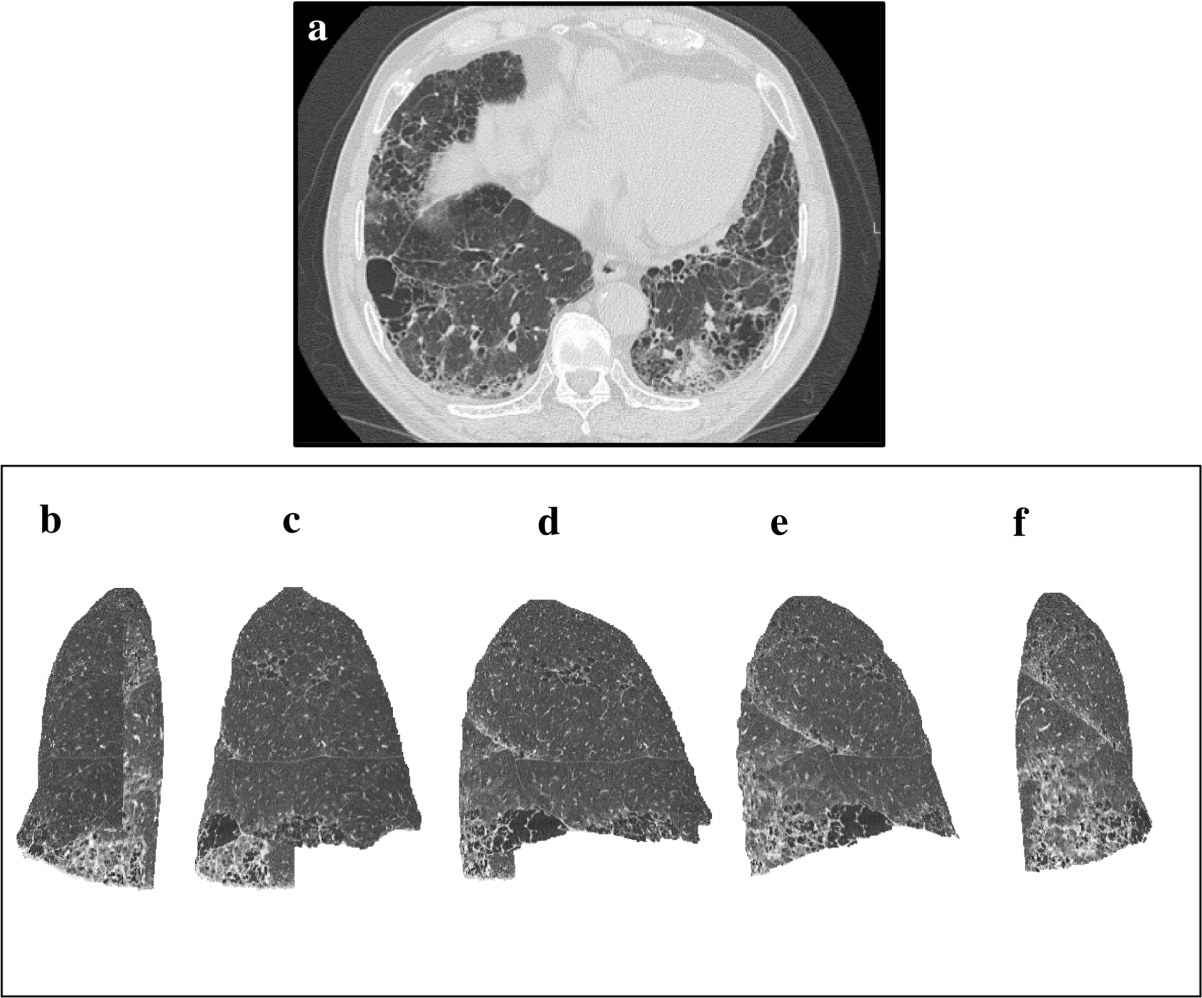
An example of HRCT axial view and 3D-cHRCT images of lung with moderate-IP. An example of HRCT axial view and 3D-cHRCT images of lung with moderate-IP. (a) HRCT axial view and 3D-HRCT image of the left lung; (b) anterior view, (c) anterolateral view, (d) right lateral view, (e) posterolateral view, (f) posterior view. %HAA values of this case was 10.34%.

Images of cases representative of each visual IP score and %HAA are shown in Figs 3–5.

### Statistical analysis

First, the study patients were divided into 3 groups according to visual IP assessment. Kappa statistics were performed to quantify the inter-reader agreement for the visual assessment of IP and emphysema. Kappa values (κ) less than 0.20 were interpreted as poor agreement, 0.21–0.40 as fair agreement, 0.41–0.60 as moderate, 0.61–0.80 as good, and 0.81–1.00 as excellent agreement. Second, the differences between the values of each group, which were obtained by CAD analysis of 3D-cHRCT images and PFT, were compared using one-way analysis of variance (ANOVA). The extent of emphysema and each UIP pattern were also assessed by Chi-square test. Two groups of patients divided according to visual IP assessment were compared using the Student *t* test. Third, multiple linear regression analysis was used to evaluate the relationship between diffusion capacity and %LAA and between diffusion capacity and %HAA. Finally, we divided patients in groups with low %DLco (values lower than 80%) and normal %DLco and used the Student *t* test to compare the differences in %LAA and %HAA between the patients with normal and those with low %DLco. Multivariate logistic regression analysis was used to assess whether or not %LAA and %HAA could be independent predictive values for low %DLco. The multivariate models simultaneously included %LAA and %HAA.

Excel 2013 software (Microsoft Corp., Redmond, WA) and SPSS version 23.0 (IBM Corp., Armonk, NY) were used for statistical analysis. *P* <0.05 was considered statistically significant.

## Results

Visual assessments of IP and emphysema by the two radiologists showed excellent consistency (κ = 0.848 and 0.806 for IP and emphysema, respectively).

Table 1 summarizes characteristics, quantitative CT measurements, and PFT values of the study patients. The study included a total of 95 patients (72 men, 23 women; age range, 45–84 years; mean age, 69 years). Eighty one patients (85.3%) had a history of smoking. There were 54 patients with emphysema. Of 48 patients with IP, 31 had mild IP and 17 had moderate IP. There were 32 patients with both emphysema and IP. Six patients suffered from connective tissue disease including rheumatoid arthritis (n = 5), and systemic sclerosis (n = 1). Only one patient had small amount of pleural effusion, and there was no patient with extensive atelectasis. The mean %LAA and %HAA values were 27.2% and 4.1%, respectively. On PFT, 25 patients had low %DLco and 70 had normal values. Volume CT dose indexes were recorded in 68 CT examinations performed after 2009, and the mean volume CT dose index was 29.8±4.6 mGy.

**Table 1:** Patient characteristics.

	Number or Mean ± SD	Range
**Patients and tumor characteristics**		
Age (years)	69.4 ± 7.3	45–84
Male / Female (n)	72 / 23	
Smoking history (n)	81	
Connective tissue disease (n)	6	
Tumor site, Right lung/Left lung (n)Right lung / Left lung	65 / 30	
**CT visual assessment**		
Goddard score 0/1/2/3/4 (n)	41 / 32 /19 / 3 / 0	
IP severity 0/1/2 (n)	47 / 31 / 17	
UIP pattern, definite/possible/inconsistent (n)	16 / 27 / 5	
**3D-cHRCT quantitative assessment**		
%LAA (%)	27.2 ± 7.6	11.8–48.6
%HAA (%)	4.1 ± 1.9	1.0–10.3
**Pulmonary function test**		
%FVC (%)	109.9 ± 17.8	63.2–148.9
%VC (%)	111.5 ± 17.6	59.1–153
FEV1/FVC (%)	70.7 ± 9.2	46.1–91.7
%DLCO (%)	101.6 ± 27.4	38.9–158.1
%DLCO/VA (%)	89.8 ± 26.3	35.0–147.5

Characterization of patients, including quantitative values.

IP, interstitial pneumonia; 3D-cHRCT, three-dimensional curved high-resolution computed tomography; UIP, usual interstitial pneumonia; %LAA, %low attenuation area; %HAA, %high attenuation area; %FVC, functional vital capacity as percent predicted; %VC, vital capacity measured as percent predicted; FEV1, forced expiratory volume in 1 second; %DLCO, diffusing capacity of the lungs for carbon monoxide measured as percent predicted; %DLCO/VA, the percent predicted diffusing capacity of the lungs for carbon monoxide divided by alveolar gas volume

Table 2 shows visual CT assessment (the extent of emphysema and UIP pattern), the mean %HAA, %LAA, and PFT values of patient groups based on visually assessed IP. The diagnosis of definite UIP pattern was seen in significantly more patients with moderate IP than in those with mild IP. The differences between the %HAA values of the 3 groups by ANOVA were significant (*P* <0.001). In addition, the mean %HAA value of the patients with moderate IP was significantly higher than the mean values of the patients without IP and the patients with mild IP (both *P* <0.001). The mean %HAA value of the patients with mild IP was significantly higher than the value of the patients without IP (*P* = 0.004). The differences between the mean %LAA values by ANOVA were not significant (*P* = 0.558). Regarding the PFT parameters, the differences in mean %DLco and %DLco/V_A_ values between the 3 groups of patients were significant (both *P* <0.001). There were no differences in the mean values of %FVC, %VC, and FEV_1_/FVC (*P* = 0.861, 0.954, and 0.586, respectively).

**Table 2:** CT visual assessment, values of %HAA and %LAA determined on 3D-cHRCT and PFT of patients classified according to visually assessed severity of interstitial pneumonia.

Visual assessment			non-IP (n = 47)	mild IP (n = 31)	moderate IP (n = 17)	*P* value
**CT visual assessment**						
	Goddard score 0/1/2/3/4 (n)		25/12/8/2/0	14/11/5/1/0	2/9/6/0/0	0.089
	UIP pattern definite/possible/inconsistent		-	2/24/5	13/2/2	<0.001
**Quantification using 3D-cHRCT**						
	%LAA		27.7 ± 8.2	27.6 ± 5.9	25.4 ± 8.7	0.558
	%HAA		3.2 ± 0.9	3.9 ± 1.2	6.9 ± 2.2	<0.001
**PFT**						
	%FVC (%)		108.9 ± 17.8	111.1 ± 17.3	110.2 ± 19.8	0.861
	%VC (%)		111.0 ± 17.1	112.1 ± 17.2	112.0 ± 17.6	0.954
	FEV1/FVC (%)		69.8 ± 9.8	71.1 ± 8.0	72.4 ± 9.6	0.586
	%DLCO (%)		112.6 ± 26.0	95.3 ± 26.9	82.8 ± 17.8	<0.001
	%DLCO/VA (%)		99.1 ± 28.2	84.5 ± 22.5	73.6 ± 15.3	<0.001

The extent of emphysema, UIP pattern, quantitative CT values (%high attenuation area [%HAA], %low attenuation area [%LAA]), and pulmonary function test (PFT) results were analyzed in 3 groups of patients classified by visual assessment of interstitial pneumonia (IP) severity. There was significant correlation between the degrees of IP and %HAA (P <0.001) and %DLCO (P <0.001). There were no significant differences in the other PFT values between the 3 types of IP.

3D-cHRCT, three-dimensional curved high-resolution computed tomography; UIP, usual interstitial pneumonia; %FVC, functional vital capacity as percent predicted;%VC, vital capacity measured as percent predicted; FEV1, forced expiratory volume in 1 second; %FEV1, forced expiratory volume in 1 second measured as percent predicted; %DLCO, diffusing capacity of the lungs for carbon monoxide measured as percent predicted; %DLCO/VA, the percent predicted diffusing capacity of the lungs for carbon monoxide divided by alveolar gas volume

**Fig 6:**
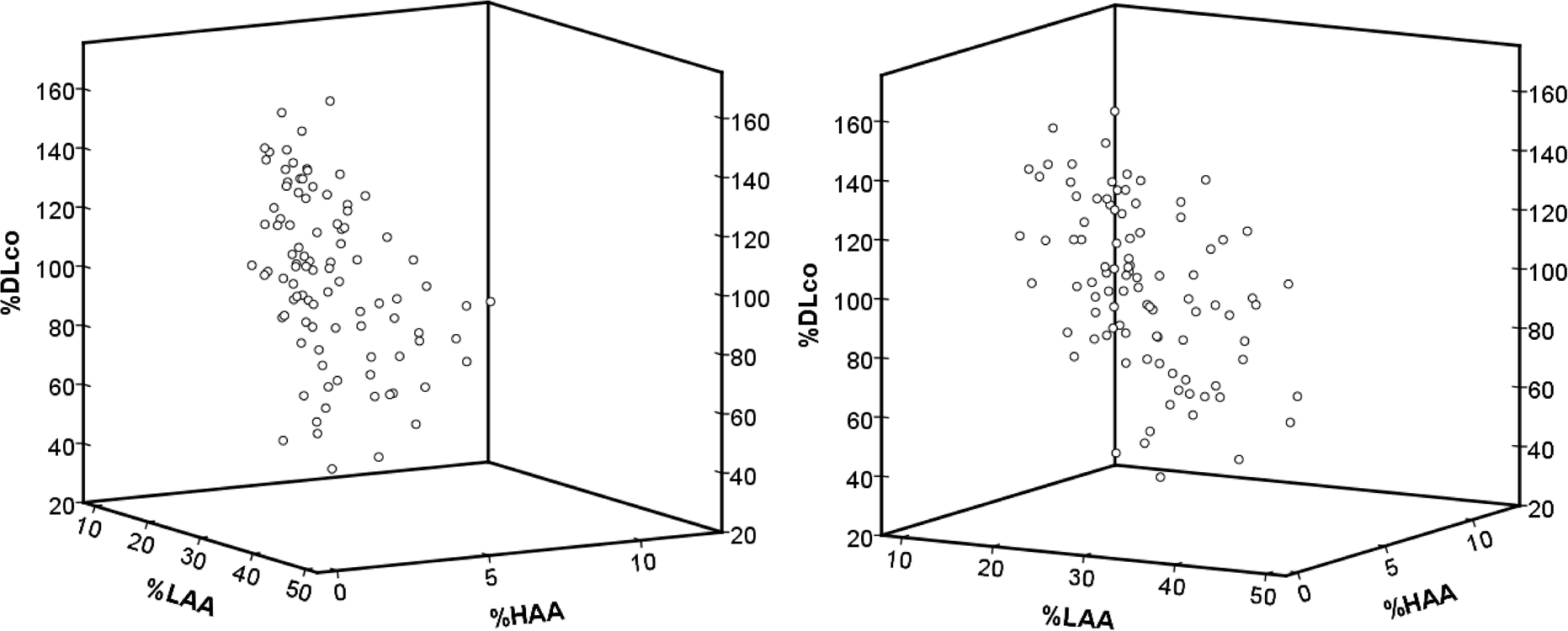
3D scatter diagrams show the correlation between %DL_CO_ and quantitative CT parameters. 3D scatter diagrams show the correlation between %DLCO and quantitative CT parameters. Both %HAA and %LAA are indicators of low diffusion capacity (standard partial regression coefficient [b*] = -0.453, *P* <0.001; b* = -0.447, P <0.001, respectively).

Table 3 shows the results of multiple linear regression analysis of the relationship between diffusion capacity and %LAA and diffusion capacity and %HAA. %HAA was significantly negatively correlated with both %DLco and %DLco/V_A_ (standard partial regression coefficient [b*] = -0.453, *P* <0.001; b* = -0.438, *P* <0.001, respectively). %LAA was also significantly negatively correlated with both %DLco and %DLco/V_A_ (b* = -0.447, *P* <0.001; b* = -0.639, *P* <0.001, respectively). Fig 6 shows the 3D scatter diagrams of %LAA, %HAA, and %DLco. Both %LAA and %HAA were negatively correlated with %DLco.

**Table 3:** Multiple linear regression analysis of diffusion capacity and attenuation values from 3D-cHRCT images.

	%DLCO		%DLCO/VA	
	Standard partial regression coefficient	*P* value	Standard partial regression coefficient	*P* value
**%LAA**	-0.447	<0.001	-0.639	<0.001
**%HAA**	-0.453	<0.001	-0.438	<0.001

Multiple linear regression analysis between diffusion capacity and attenuation values determined from three-dimensional curved high-resolution computed tomography (3D-cHRCT) images suggests that both %high attenuation area (%HAA) and %low attenuation area (%LAA) are correlated with diffusion capacity.

%DLCO, diffusing capacity of the lungs for carbon monoxide measured as percent predicted; %DLCO/VA, the percent predicted diffusing capacity of the lungs for carbon monoxide divided by alveolar gas volume

Table 4 compares the mean %LAA and %HAA values of the patients with normal and low diffusion capacity. The mean %HAA of patients with low %DLco was significantly higher than the mean %HAA of patients with normal %DLco (*P* = 0.011). The mean %LAA of patients with low %DLco was higher than the mean %LAA of the patients with normal %DLco, although the difference was not significant (*P* = 0.080). Multivariate logistic regression analysis found that both %LAA and %HAA were significantly associated with risk of low %DLco (Table 5; OR = 1.111, *P* = 0.006; OR = 1.614, *P* = 0.002, respectively).

**Table 4:** Attenuation values from 3D-cHRCT images of patients with normal and low %DLCO.

	Normal %DLCO ≥80%	Low %DLCO <80%	*P* value
**Number**	70	25	
**%LAA**	26.6 ± 7.2	29.1 ± 8.7	0.080
**%HAA**	3.9 ± 1.5	4.8 ± 2.5	0.011

Comparisons of %low attenuation area (%LAA) and %high attenuation area (%HAA) in patients with normal and low diffusion capacity. Both %HAA and %LAA of patients with low diffusing capacity of the lungs for carbon monoxide measured as percent predicted (%DLco) were higher than the values of the patients with normal %DLco. However, only %HAA was significantly different (*P* = 0.011).

3D-cHRCT, three-dimensional curved high-resolution computed tomography; %DLCO, diffusing capacity of the lungs for carbon monoxide measured as percent predicted

**Table 5:** Multivariate logistic regression analysis for %DLCO <80%.

Variables	Odds Ratio	95% CI	*P* value
**%LAA**	1.111	1.031–1.199	0.006
**%HAA**	1.614	1.202–2.169	0.002

Multivariate logistic regression analysis for %DLCO <80% was performed. The multivariate models simultaneously included %LAA and %HAA. Both %low attenuation area (%LAA) and %high attenuation area (%HAA) were significant indicators of impaired diffusion capacity (OR = 1.111, *P* = 0.006; OR = 1.614, *P* = 0.002, respectively).

%DLCO, diffusing capacity of the lungs for carbon monoxide measured as percent predicted; CI, confidence interval

## Discussion

In this retrospective study, we investigated the association between the %HAA and %LAA values that were computed using 3D-cHRCT and a new CAD and the diffusion capacity in patients with lung cancer. Our results demonstrated the following 3 points. 1) The %HAA and diffusion capacity were significantly correlated with the visual IP grades of non-IP, mild IP, and moderate IP. 2) Both the %HAA and the %LAA were significantly correlated with %DLco, according to multiple linear regression analysis. 3) Both the %HAA and the %LAA were independent predictors for impaired diffusion capacity by multivariate analysis. These findings suggest that the %HAA and the %LAA obtained from 3D-cHRCT images of the lung at a 1-cm depth from the chest wall might be useful for the quantification of IP.

HRCT is essential for diagnosing and determining the extent of diffuse interstitial lung disease [4]. HRCT is also an important tool for predicting the clinical outcomes of patients with idiopathic pulmonary fibrosis (IPF), because the extent of fibrosis seen on HRCT correlates well with mortality rate[13, 14]. The use by radiologists of visual assessment of fibrosis is popular, but even experienced radiologists are subject to intra- and interobserver error due to misinterpretation of HRCT patterns [13].

Some computed IP quantification systems have been developed [14–21]. Many evaluate the entire lung field and quantify each pattern of abnormalities (i.e., consolidation, honeycombing, ground-glass opacity, etc.). However, evaluation of the entire lung field can be complicated; and sometimes manual contouring is needed, especially for bilateral hilar lesions, where there are thick bronchi and vessels. By visual assessment, expert radiologists use their knowledge and experience to differentiate pathognomonic IP changes from the normal underlying anatomic landmarks. However, current CAD methods cannot automatically distinguish them, because both pathognomonic and structural features appear as areas of high attenuation on HRCT. Significant variation in the lung density of normal individuals also leads to difficulty in quantifying IP infiltrates and inaccurate results [22].

To resolve these difficulties, we developed a novel quantification system that only targets the peripheral field. Our system should be an easier method to use than the quantification systems used in previous reports. In patients with idiopathic IP, especially those with UIP and non-specific interstitial pneumonia, fibrotic changes such as reticular pattern, ground-glass opacity, and honeycombing, show peripheral and basal dominance even during the early stages of disease, and these changes are most severe in the subpleural parenchyma [23]. On the other hand, thick vessels or bronchi are rare in peripheral lung.

We theorized that our novel 3D-imaging of the peripheral lung field only should minimize the confounding effects of normal lung structures, and highlight the %HAA representing fibrotic change. As expected, the %HAA on 3D-cHRCT was significantly correlated with visual assessment of IP and PFT diffusion capacity. 3D-cHRCT could simultaneously quantify emphysema as %LAA. Therefore, these parameters as assessed on 3D-cHRCT images are sensitive to the severity of both fibrotic changes and emphysema.

In this study, we adopted -500 HU as the threshold value of %HAA. Results of a preliminary experiment suggested that -500 HU was the best indicator of diffusion capacity. Our result is consistent with the report of Sverzellati1 *et al*. that indicated that pixels greater than -500 HU were representative of lung fibrosis and inflammation [11]. On the other hand, we adopted -950 HU as the threshold value of %LAA. Areas with attenuation values lower than -950 HU had good histopathological correlation with emphysema [24], and %LAA has been used as an indicator of emphysema [7, 8]. Honeycomb cysts in UIP/IPF or cysts in airspace enlargement with fibrosis were also recognized as LAA, which cannot differentiate from emphysema. However, the extent of cystic dead space contributes to the lack of perfusion in the fibrotic lung and is linked to reduction in DLco. These suggest that both parameters together, %HAA and %LAA, would be a better indicator of low diffusion capacity than each value by itself.

Our method of imaging and assessment is also useful for evaluating combined pulmonary fibrosis and emphysema (CPFE). IP, emphysema, and CPFE are often comorbidities of lung cancer, because these diseases are closely related to a history of smoking. IP, emphysema, and CPFE sometimes affect the treatment strategy used for lung cancer [25]. Many studies have found that FVC and FEV_1_ are preserved in patients with CPFE, as compared to those with either IP or Chronic obstructive pulmonary disease [3]. For this reason, PFT that includes measurement of DLco is needed for clinical evaluation of patients who might have CPFE. Our results suggest that we could estimate the diffusion capacity of the lung on curved MPR images obtained by routine HRCT. Therefore, it may be a screening test to detect CPFE patients who need further PFT including DLco.

There are several limitations to this study. First, it was a retrospective and single-center study. Second, all cases were recruited from a lung cancer database, and we did not study the effects of lung cancer. However, lung cancer patients who are candidates for surgery often undergo preoperative HRCT and PFT during the same time period, and we could easily acquire clinical data. In addition, patients with lung cancer frequently have a positive smoking history, and many have comorbid emphysema and IP. Third, the diagnosis of IP was based on radiological findings, not on the histological examination. We can diagnose typical idiopathic pulmonary fibrosis based on HRCT findings only, but we could not definitively diagnose atypical IPF or other types of IP. Actually, not only CT findings but also clinical information should be taken into consideration for proper diagnosis [26]. Fourth, the patients in this study were all pending surgical procedures, and patients who could not take surgical resection because of severe IP were not included. We need further study to determine whether this CAD is applicable for patients with severe IP.

## Conclusions

The %HAA and %LAA values computed using a novel CAD to assess a 3D-cHRCT images at a constant depth from the chest wall were significantly correlated not only with IP visual assessments but also with DLco, and may be novel and important quantitative parameters for IP, emphysema, and CPFE comorbid with lung cancer.

## Supporting information

S1 Table Comparison of quantification using 3D-cMPR obtained with various depth and thresholds between normal and low diffusion capacity group. Quantification of high-attenuation area (HAA) values obtained from 3D-cHRCT images at varying depths from the chest wall, and HU thresholds according to patients with low and normal diffusing lung capacity of carbon monoxide measured as percent predicted (%DLCO). The table suggests that %high attenuation area (%HAA) > -500HU derived from 3D-cHRCT images at 1-cm depth has a better indication of %DLCO as far as we investigated. 3D-cHRCT, three-dimensional curved high-resolution computed tomography; %LAA: %low attenuation area.
(PPTX)
