# Supplementary material for: Quantitative evaluation of interstitial pneumonia using 3D-curved high-resolution CT imaging parallel to the chest wall: A pilot study
Source: PLoS One. 2017 Sep 28;12(9):e0185532. doi: 10.1371/journal.pone.0185532 (PMC5619786; doi:10.1371/journal.pone.0185532)
Supplement: S1 Table — Quantification of high-attenuation area (HAA) values obtained from 3D-cHRCT images at varying depths from the chest wall, and HU thresholds according to patients with low and normal diffusing lung capacity of carbon monoxide measured as percent predicted (%DLCO). The table suggests that %high attenuation area (%HAA) > -500HU derived from 3D-cHRCT images at 1-cm depth has a better indication of %DLCO as far as we investigated. 3D-cHRCT, three-dimensional curved high-resolution computed tomography; %LAA: %low attenuation area. (PPTX) [file pone.0185532.s001.pptx]

## Slide 1
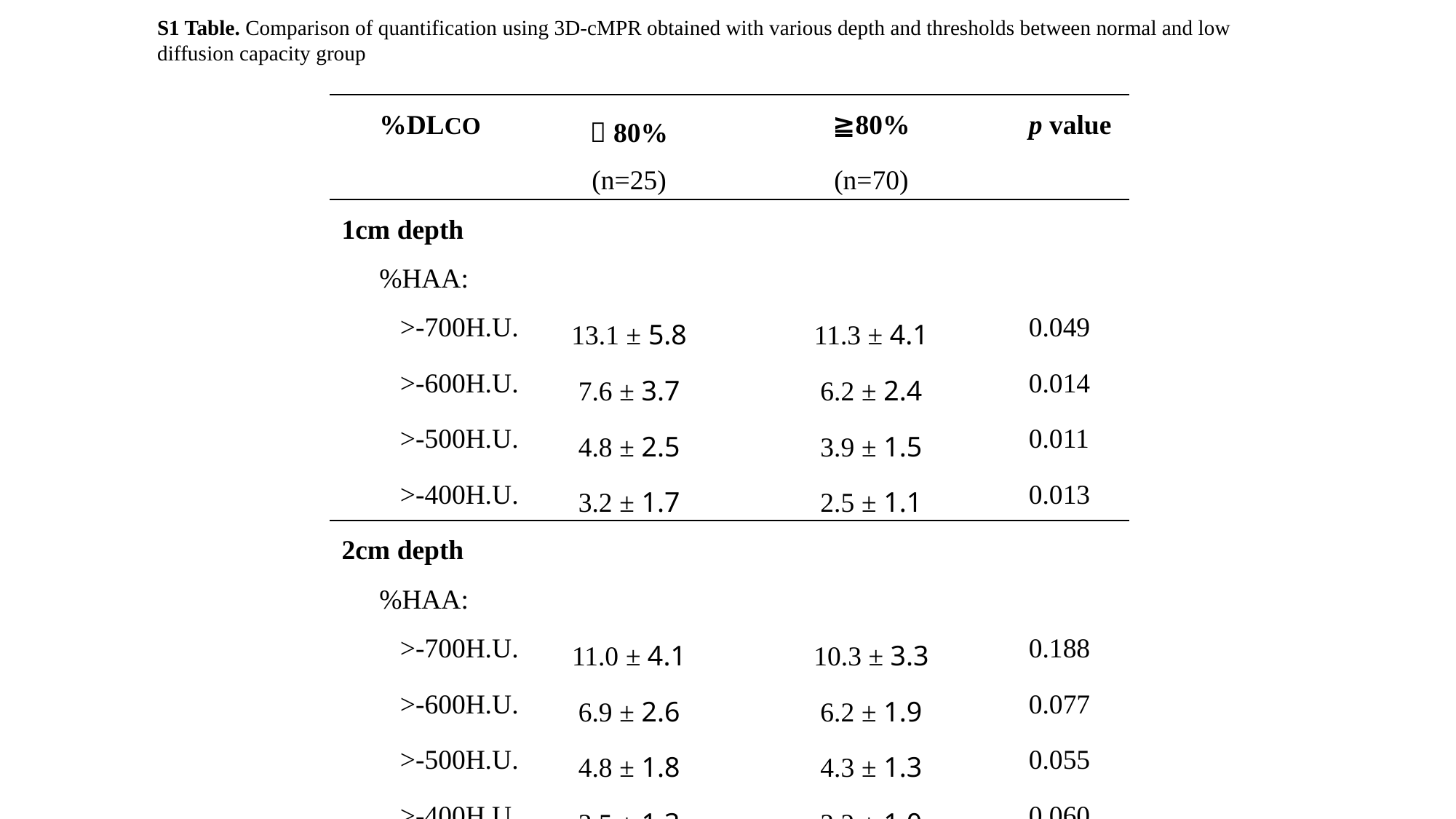

S1 Table. Comparison of quantification using 3D-cMPR obtained with various depth and thresholds between normal and low diffusion capacity group
| | %DLCO | ＜80% | | | | ≧80% | | | | p value |
| --- | --- | --- | --- | --- | --- | --- | --- | --- | --- | --- |
| | | (n=25) | | | | (n=70) | | | | |
| 1cm depth | | | | | | | | | | |
| | %HAA: | | | | | | | | | |
| | >-700H.U. | 13.1 ± 5.8 | | | | 11.3 ± 4.1 | | | | 0.049 |
| | >-600H.U. | 7.6 ± 3.7 | | | | 6.2 ± 2.4 | | | | 0.014 |
| | >-500H.U. | 4.8 ± 2.5 | | | | 3.9 ± 1.5 | | | | 0.011 |
| | >-400H.U. | 3.2 ± 1.7 | | | | 2.5 ± 1.1 | | | | 0.013 |
| 2cm depth | | | | | | | | | | |
| | %HAA: | | | | | | | | | |
| | >-700H.U. | 11.0 ± 4.1 | | | | 10.3 ± 3.3 | | | | 0.188 |
| | >-600H.U. | 6.9 ± 2.6 | | | | 6.2 ± 1.9 | | | | 0.077 |
| | >-500H.U. | 4.8 ± 1.8 | | | | 4.3 ± 1.3 | | | | 0.055 |
| | >-400H.U. | 3.5 ± 1.3 | | | | 3.2 ± 1.0 | | | | 0.060 |
